# Phenotyping cardiogenic shock that showed different clinical outcomes and responses to vasopressor use: a latent profile analysis from MIMIC-IV database

**DOI:** 10.3389/fmed.2023.1186119

**Published:** 2023-06-22

**Authors:** Yue Yu, Jin Rao, Qiumeng Xu, Jian Xiao, Pengchao Cheng, Junnan Wang, Wang Xi, Pei Wang, Yufeng Zhang, Zhinong Wang

**Affiliations:** ^1^Department of Cardiothoracic Surgery, Changzheng Hospital, Naval Medical University, Shanghai, China; ^2^Department of Orthopaedics, Changzheng Hospital, Naval Medical University, Shanghai, China

**Keywords:** cardiogenic shock, phenotype, latent profile analysis, vasopressor, mortality

## Abstract

**Background:**

Cardiogenic shock (CS) is increasingly recognized as heterogeneous in its severity and response to therapies. This study aimed to identify CS phenotypes and their responses to the use of vasopressors.

**Method:**

The current study included patients with CS complicating acute myocardial infarction (AMI) at the time of admission from the Medical Information Mart for Intensive Care IV (MIMIC-IV) database. Laboratory and clinical variables were collected and used to conduct latent profile (LPA) analysis. Furthermore, we used a multivariable logistic regression (LR) model to explore the independent association between the use of vasopressors and endpoints.

**Result:**

A total of 630 eligible patients with CS after AMI were enrolled in the study. The LPA identified three profiles of CS: profile 1 (*n* = 259, 37.5%) was considered as the baseline group; profile 2 (*n* = 261, 37.8%) was characterized by advanced age, more comorbidities, and worse renal function; and profile 3 (*n* = 170, 24.6%) was characterized by systemic inflammatory response syndrome (SIRS)-related indexes and acid–base balance disturbance. Profile 3 showed the highest all-cause in-hospital mortality rate (45.9%), followed by profile 2 (43.3%), and profile 1 (16.6%). The LR analyses showed that the phenotype of CS was an independent prognostic factor for outcomes, and profiles 2 and 3 were significantly associated with a higher risk of in-hospital mortality (profile 2: odds ratio [OR] 3.95, 95% confidence interval [CI] 2.61–5.97, *p* < 0.001; profile 3: OR 3.90, 95%CI 2.48–6.13, *p* < 0.001) compared with profile 1. Vasopressor use was associated with an improved risk of in-hospital mortality for profile 2 (OR: 2.03, 95% CI: 1.15–3.60, *p* = 0.015) and profile 3 (OR: 2.91, 95% CI: 1.02–8.32, *p* = 0.047), respectively. The results of vasopressor use showed no significance for profile 1.

**Conclusion:**

Three phenotypes of CS were identified, which showed different outcomes and responses to vasopressor use.

## Introduction

Cardiogenic shock (CS) is defined as a state of reduced cardiac output due to a primary cardiac problem that results in end-organ hypoperfusion (1). The most common etiology of CS is acute myocardial ischemia (AMI), accounting for up to 80% of the cases (2). Although concepts have evolved and management has advanced, CS-related mortality remains unexpectedly high (3–6). There have been numerous clinical trials aiming at testing whether certain drugs (for example, vasopressor and inodilator) or other interventions might reduce mortality but showing conflicting results (1, 6–9). One of the possible reasons for the failure of these trials to identify positive outcomes was the case-mix problem. CS encompasses a heterogeneous population in terms of underlying causes, clinical characteristics, outcomes, and possibly treatment response (10).

Therefore, individualized patient care is recommended to improve survival outcomes (11). The goal of individualized medicine is to tailor healthcare to each patient by identifying phenotypes of patients who present with distinct clinical characteristics and respond to individualized treatments. However, most attempts at staging CS have been based on expert opinions and consensus (10, 12–14). To avoid complexity, some of these classification systems only use few variables and depend on specific, although arbitrary, cutoffs, which may introduce bias and fail to capture the full variability of patient profiles (15). Additionally, many previous studies include unselected CS patients, and the etiology of CS has not been identified accurately, which might lead to result bias (12, 16). Furthermore, several previous studies did not explore the relationship between treatment effects and outcomes in different phenotypes (2, 12, 17).

To address the above issues and overcome the shortcomings of previous studies, the purpose of the study was to determine whether phenotypes could be identified using latent profile analysis (LPA) by analyzing routinely collected clinical data. The identified profiles’ responses to widely used vasopressors were also compared.

## Methods

### Study design, database, and ethics approval

Based on the methods used in our previously published studies (18–22), we conducted a retrospective analysis using all the relevant data extracted from the Medical Information Mart for Intensive Care IV (MIMIC-IV version 2.0) database (23). The MIMIC-IV database is an updated version of MIMIC-III and currently contains comprehensive and high-quality data of patients admitted to intensive care units (ICUs) at the Beth Israel Deaconess Medical Center between 2008 and 2022. An individual who has finished the Collaborative Institutional Training Initiative examination (Certification number: 33281932) can access the database. The establishment of the MIMIC-IV database was approved by the Massachusetts Institute of Technology and the Institutional Review Board of Beth Israel Deaconess Medical Center (BIDMC) (24). Prior to study analyses, all data were made anonymous, so informed consent was not necessary.

### Study population

We included all critically ill patients with a primary diagnosis of CS after AMI using diagnostic codes of the International Classification of Diseases, 9th revised (ICD-9) and 10th revised (ICD-10) editions (ICD codes of CS: 78551 and R570; ICD codes of AMI: 410, 411, 412, I21, I22, and I252). Patients were excluded if they had: (1) the age of less than 18; (2) multiple ICU admissions; (3) a length of stay in the ICU less than 24 h; and (4) incomplete information about study outcome and treatment-related data.

### Variables and endpoints

Since our goal was to phenotype CS patients based on available data at the time of ICU admission, we only used data that was available within 24 h of ICU admission for further analyses. On the first day of admission, only the initial test results were taken into account for subsequent analyses if patients had received multiple measurements of their vital signs or laboratory tests.

The extracted variable included: (1) demographics: age, gender, and race; (2) admission type (urgent, emergency, and other); (3) vital signs: systolic blood pressure (SBP), diastolic blood pressure (DBP), mean blood pressure (MBP), heart rate, respiratory rate, temperature, and urine output; (4) laboratory findings: white blood cell (WBC) count, red blood cell (RBC) count, platelet count, red blood cell distribution width (RDW), blood urea nitrogen (BUN), creatinine, estimated glomerular filtration rate (eGFR), glucose, bilirubin, alanine aminotransferase, total calcium, potassium, sodium, chloride, and bicarbonate, pH, arterial oxygen partial pressure (P_a_O_2_), partial pressure of carbon dioxide (PCO_2_), anion gap, base excess, lactate, activated partial thromboplastin time (aPTT), and international normalized ratio (INR); The eGFR was calculated using the modification of diet in renal disease (MDRD) formula (25); (5) prognostic scoring system: systemic inflammatory response syndrome (SIRS) score and Charlson comorbidity score; (6) Treatment information: intra-aortic balloon pump (IABP), mechanical ventilation, renal replacement treatment (RRT), and vasopressors (dopamine, epinephrine, or norepinephrine). All vasopressors were administered via continuous infusion.

The endpoints of our study included all-cause in-hospital and ICU mortality, length of stay (LOS) in ICU and hospital, duration of ventilation, and use of IABP and RRT.

### Missing values

Due to the scarcity of the data samples, the missing variables were not simply eliminated. Instead, multiple imputation with chained equations, based on five replications, was used to account for missing data. Variables (body mass index, total protein, albumin, fraction of inspired oxygen, neutrophils, and oxygen saturation in the arterial blood) have values greater than 20% and were excluded from the study. The remaining variables were subjected to multiple imputation (26).

### Latent profile analysis

The goal of LPA, a type of unsupervised machine learning method, is to uncover hidden groups or patterns in observed data (27). The observed data in the current study were CS patients’ vital signs and laboratory tests taken within the first 24 h of being admitted to the ICU, while the hidden groups were latent subphenotypes of CS. The distributions of the included variables were examined prior to analysis, and severely skewed data would be transformed. Bootstrap likelihood ratio test (BLRT), Bayesian information criteria (BIC), the number of people in each class, and the clinical interpretation were used in the current study to determine the optimal number of profiles (28, 29). BIC was utilized to compare models with different cluster numbers or specifying different parameterizations or both. Better model fit is indicated by lower BIC values (30). The number of mixture components in a particular finite mixture model parameterization was evaluated using BLRT. The observed significance is estimated using the bootstrap for the likelihood ratio test statistic. *p* values were calculated by BLRT for the k-class model versus (k-1)-class model comparison (29). The BLRT’s statistical importance was determined using a *p* value threshold of 0.05. Additionally, we pre-specified that the patient proportion in any of the latent profiles should be greater than 5%, as each latent profile should have a sizable number of patients (31). The underlying subphenotypes were also confirmed utilizing latent class analysis (LCA) (32). To visualize the optimal phenotype results and patterns in clinical variables, the data were analyzed with ranked plots of the variables by the mean and standardized difference among the phenotypes.

### Statistical analysis

For continuous variables, values are shown as the mean (standard deviation [SD]) or median (interquartile range [IQR]) as appropriate. For categorical variables, the total number (percentage) is presented. Comparisons between groups were made using the Chi-Squared test or Fisher’s exact-test for categorical variables and the analysis of variance for continuous variables as appropriate.

After adjusting for some covariates (gender, race, and admission type), multivariable logistic regression (LR) was used to determine whether endpoints varied between profiles. Additionally, to investigate the independent influence of CS profile on outcomes, the LR model was also employed to investigate the relationship between vasopressor and outcomes in each profile.

All statistical analyses were performed using R software (version 4.1.2). Two-tailed *p* values less than 0.05 were considered statistically significant.

## Results

### Patient and variable selection

**Figure 1:**
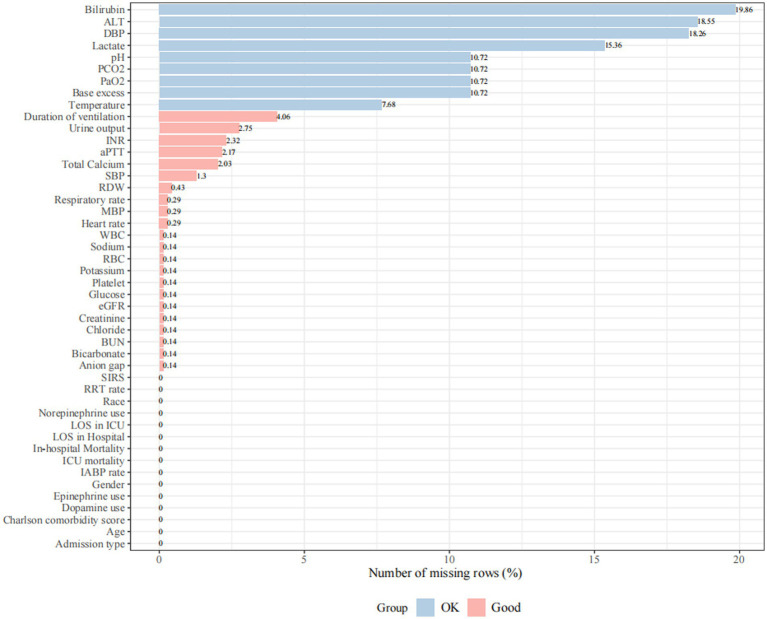
Missing rate for clinical and laboratory variables was extracted from the database. Missing rate < 5% is defined as “Good” and 5% ≤ missing rate ≤ 20% is defined as “OK”. Variables with a missing rate greater than 20% (body mass index, total protein, albumin, fraction of inspired oxygen, neutrophils, and oxygen saturation in the arterial blood) were excluded from the analysis. SBP, systolic blood pressure; DBP, diastolic blood pressure; MBP, mean blood pressure; WBC, white blood cell; RBC, red blood cell; RDW, red blood cell distribution width; BUN, blood urea nitrogen; eGFR, estimated glomerular filtration rate; PaO_2_, arterial oxygen partial pressure; PCO_2_, partial pressure of carbon dioxide; aPTT, activated partial thromboplastin time; INR, international normalized ratio; SIRS, systemic inflammatory response syndrome; IABP, intra-aortic balloon pump; RRT, renal replacement treatment; LOS, length of stay; ALT, alanine aminotransferase.

We initially identified 76,540 records of ICU admission from the MIMIC-IV 2.0 database. After the application of inclusion and exclusion criteria, a total of 690 CS patients after AMI were included for analysis (Figure 1). A total of 33 continuous variables were incorporated into the mode.

### Choose the best number of latent profiles

Models with different number of profiles were compared. Supplementary Table S1 illustrates the statistics for choosing the most appropriate number of profiles. The 3-profile model showed the lowest values in BIC (67,296.57). The three-profile model had a sizable number of patients in each profile (259, 261, and 170 for profiles 1, 2, and 3, respectively). When there were four or more profiles, some profiles’ patient counts differed by less than 5% from those of other profiles. The three-profile model was deemed the best model when all profile selection criteria were taken into account.

### Different clinical features between profiles

**Figure 2:**
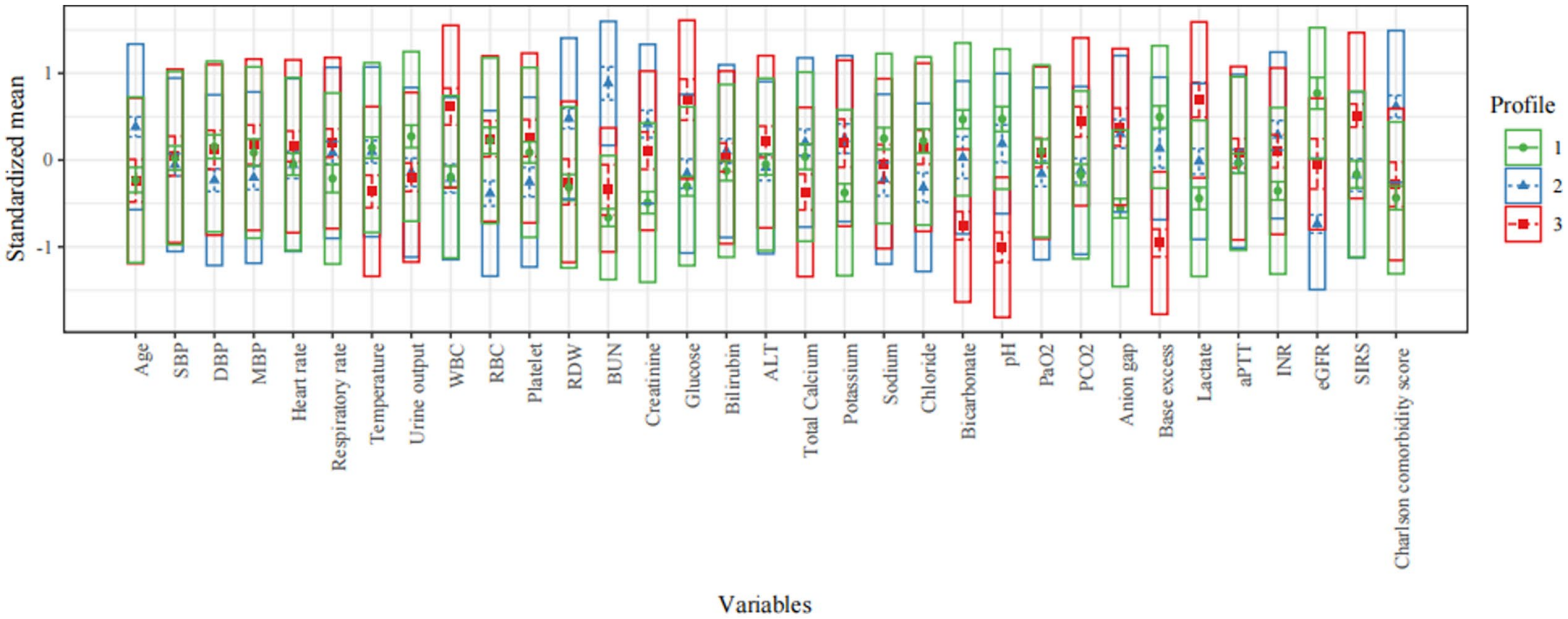
Characteristics of latent profile groups. The y-axis shows the standardized mean for each variable (that is, each variable is centered at the sample mean and scaled by its standard deviation). SBP, systolic blood pressure; DBP, diastolic blood pressure; MBP, mean blood pressure; WBC, white blood cell; RBC, red blood cell; RDW, red blood cell distribution width; BUN, blood urea nitrogen; eGFR, estimated glomerular filtration rate; PaO_2_, arterial oxygen partial pressure; PCO_2_, partial pressure of carbon dioxide; aPTT, activated partial thromboplastin time; INR, international normalized ratio; SIRS, systemic inflammatory response syndrome; ALT, alanine aminotransferase.

Clinical features of all three profiles are shown in Tables 1, 2 and Figure 2. Profile 1 (*n* = 259, 37.5%) was considered as the baseline profile to compare with other profiles. Profile 2 had the largest proportion of all CS patients (*n* = 261, 37.8%) and was characterized by more advanced age, more comorbidities (Charlson comorbidity score), and worse renal function(creatinine, BUN, and eGFR). Profile 3 (*n* = 170, 24.6%) was characterized by SIRS-related indexes (SIRS score, glucose, WBC, and respiratory rate) and acid–base balance disturbance (bicarbonate, pH, base excess, lactate, and PCO_2_).

**Table 1:** Continuous variables included in the latent profile analysis.

Characteristics	Profile 1 (*n = 259*)	Profile 2 (*n = 261*)	Profile 3 (*n = 170*)	*p*
*Demographics*				
Age, year	69.6 [60.2; 77.9]	78.6 [69.7; 85.8]	69.7 [61.0; 78.1]	<0.001
*Vital signs*				
SBP, mmHg	113 [101; 126]	111 [95.0; 126]	114 [100; 132]	0.285
DBP, mmHg	67.0 [55.0; 79.0]	62.0 [52.2; 73.8]	72.0 [62.0; 86.0]	<0.001
MBP, mmHg	83.0 [70.0; 92.0]	76.0 [67.0; 87.0]	82.0 [71.2; 96.8]	<0.001
Heat rate, beats/min	87.0 [76.0; 101]	87.0 [73.0; 101]	89.5 [81.0; 110]	<0.001
Respiratory rate, beats/min	19.0 [16.0; 22.0]	20.0 [17.0; 25.0]	22.0 [18.0; 25.0]	<0.001
Temperature, ◦C	36.7 [36.4; 36.9]	36.6 [36.3; 36.9]	36.4 [35.7; 36.7]	<0.001
Urine output, L/24 h	1930 [1155; 3064]	1135 [479; 2340]	1296 [620; 2191]	<0.001
*Laboratory findings*				
WBC, 10^9^/L	12.2 [8.50; 15.9]	11.9 [9.17; 14.7]	16.9 [13.1; 21.9]	<0.001
RBC, 10^9^/L	4.09 (0.81)	3.56 (0.77)	4.09 (0.80)	<0.001
Platelet, 10^9^/L	225 [180; 293]	192 [147; 252]	236 [175; 316]	<0.001
RDW, %	13.8 [13.1; 15.0]	15.4 [14.4; 17.1]	13.9 [13.2; 15.2]	<0.001
BUN, mg/dL	20.0 [15.0; 27.0]	50.5 [39.0; 68.0]	25.0 [19.0; 31.8]	<0.001
Creatinine, mg/dL	1.00 [0.90; 1.20]	2.10 [1.67; 3.23]	1.40 [1.10; 1.98]	<0.001
eGFR, mL/min/1.73m^2^	66.4 [52.0; 83.2]	25.4 [17.2; 34.6]	42.7 [30.9; 53.9]	<0.001
Glucose, mg/dL	150 [120; 188]	153 [118; 206]	258 [180; 365]	<0.001
Bilirubin, mg/dL	0.60 [0.40; 1.00]	0.80 [0.50; 1.40]	0.70 [0.40; 1.20]	0.004
ALT	43.0 [25.0; 95.0]	47.0 [22.0; 125]	95.0 [47.0; 243]	<0.001
Total Calcium, mmol/L	8.40 [8.00; 8.90]	8.50 [8.20; 9.00]	8.10 [7.50; 8.50]	<0.001
Potassium, mmol/L	4.10 [3.80; 4.50]	4.60 [4.20; 5.20]	4.50 [4.00; 5.20]	<0.001
Sodium, mmol/L	139 [136; 141]	136 [133; 140]	137 [134; 140]	<0.001
Chloride, mmol/L	103 [100; 106]	99.0 [94.0; 104]	103 [99.0; 106]	<0.001
Bicarbonate, mmol/L	23.0 [20.0; 25.0]	20.0 [18.0;24.0]	17.0 [14.0; 19.0]	<0.001
pH	7.39 [7.34; 7.43]	7.35 [7.30;7.41]	7.22 [7.15; 7.27]	<0.001
PaO_2_	95.0 [63.0; 195]	76.0 [41.0;148]	83.5 [52.0; 137]	0.001
PCO_2_	40.0 [34.0; 45.0]	40.0 [33.0;45.0]	46.0 [38.0; 54.0]	<0.001
Anion gap	15.0 [13.0; 17.0]	19.0 [16.0;22.0]	20.0 [17.0; 24.0]	<0.001
Base excess	−1.00 [−3.00;0.00]	−3.00 [−6.00;0.00]	−9.00 [−12.00; −7.00]	<0.001
Lactate	1.80 [1.30; 2.40]	2.20 [1.58; 3.80]	4.30 [2.40; 7.00]	<0.001
aPTT	38.8 [28.5; 60.3]	38.1 [30.0; 64.4]	45.8 [30.7; 89.6]	0.004
INR	1.20 [1.10; 1.40]	1.40 [1.20; 2.00]	1.30 [1.20; 1.78]	<0.001
*Prognostic scoring system*				
SIRS	3.00 [2.00;3.00]	3.00 [2.00;3.00]	3.00 [3.00;4.00]	<0.001
Charlson comorbidity score	6.00 [5.00;8.00]	9.00 [8.00;10.0]	7.00 [6.00;8.00]	<0.001

SBP, systolic blood pressure; DBP, diastolic blood pressure; MBP, mean blood pressure; WBC, white blood cell; RBC, red blood cell; RDW, red blood cell distribution width; BUN: blood urea nitrogen; eGFR: estimated glomerular filtration rate; PaO_2_: arterial oxygen partial pressure; PCO_2_: partial pressure of carbon dioxide; aPTT: activated partial thromboplastin time; INR: international normalized ratio; SIRS: systemic inflammatory response syndrome; ALT: alanine aminotransferase.

**Table 2:** Categorical variables and outcome/treatment variables not included in the latent profile analysis.

Characteristics	Profile 1 (*n = 259*)	Profile 2 (*n = 261*)	Profile 3 (*n = 170*)	*p*
Male gender	164 (63.3%)	162 (62.1%)	106 (62.4%)	0.954
Race				0.003
Black	14 (5.41%)	12 (4.60%)	6 (3.53%)	
White	178 (68.7%)	190 (72.8%)	97 (57.1%)	
Other	67 (25.9%)	59 (22.6%)	67 (39.4%)	
Admission type				0.002
Urgent	125 (48.3%)	109 (41.8%)	63 (37.1%)	
Emergency.	92 (35.5%)	99 (37.9%)	89 (52.4%)	
Other	42 (16.2%)	53 (20.3%)	18 (10.6%)	
Vasopressor use				
Dopamine use	54 (20.8%)	65 (24.9%)	62 (36.5%)	0.001
Epinephrine use	40 (15.4%)	27 (10.3%)	41 (24.1%)	0.001
Norepinephrine use	14 (44.0%)	146 (55.9%)	125 (73.5%)	<0.001
Endpoints				
LOS in ICU, h	97.5 [64.6;178]	98.2 [54.3;166]	131 [74.1;234]	0.001
LOS in Hospital, h	237 [142;402]	235 [123;411]	234 [115;351]	0.278
Duration of ventilation, h	3.8 [33.5;133]	64.2 [33.0;114]	97.7 [56.0;172]	<0.001
IABP	44 (17.0%)	30 (11.5%)	28 (16.5%)	0.163
RRT	10 (3.86%)	64 (24.5%)	44 (25.9%)	<0.001
In-hospital Mortality	43 (16.6%)	113 (43.3%)	78 (45.9%)	<0.001
ICU mortality	28 (10.8%)	88 (33.7%)	68 (40.0%)	<0.001

IABP, intraaortic balloon pump; RRT, renal replacement treatment; LOS, length of stay; ICU, intensive care unit.

### Association between profiles with endpoints

Compared with patients in the other two profiles (profiles 1 and 2), patients in profile 3 had the longest length of stay in ICU (median: 131; IQR: 74 to 234 h) and duration of ventilation (median: 97.7; IQR: 56.0–172 h; Table 2). Additionally, patients in profile 3 had the highest use of IABP rate (16.5%), use of RRT rate (25.9%), in-hospital and ICU mortality rate (45.9 and 40.0%), followed by patients in profile 2 and profile 1. Multivariable LR analyses demonstrated that in-hospital mortality differed significantly among the three latent profiles (Table 3). Compared to the profile 1, profiles 2 was 3 were significantly associated with a higher risk of in-hospital mortality (profile 2: odds ratio [OR] 3.95, 95% confidence interval [CI] 2.61–5.97, *p* < 0.001; profile 3: OR 3.90, 95%CI 2.48–6.13, *p* < 0.001) and ICU mortality (profile 2: OR 4.43, 95%CI 4.43, *p* < 0.001; profile 3: OR 5.14, 95%CI 3.09–8.52, *p* < 0.001) (Table 3).

**Table 3:** The associations of profiles and endpoints.

	Hospital mortality	ICU mortality				
	OR	95%CI	*p*	OR	95%CI	*p*
Profile 1	Ref			Ref		
Profile 2	3.95	2.61-5.97	<0.001	4.43	2.75–7.11	<0.001
Profile 3	3.90	2.48-6.13	<0.001	5.14	3.09–8.52	<0.001
Interaction between profile and vasopressor						
The whole cohort	1.99	1.36-2.90	<0.001	3.36	2.12–5.35	<0.001
Profile 1	1.18	0.59-2.36	0.642	1.45	0.62–3.38	0.385
Profile 2	2.03	1.15-3.6	0.015	3.61	1.82–7.14	<0.001
Profile 3	2.91	1.02-8.32	0.047	5.57	1.51–20.52	0.010

OR, odds ratio; CI, confidence interval; ICU, intensive care unit.

### Sensitivity analysis

**Figure 3:**
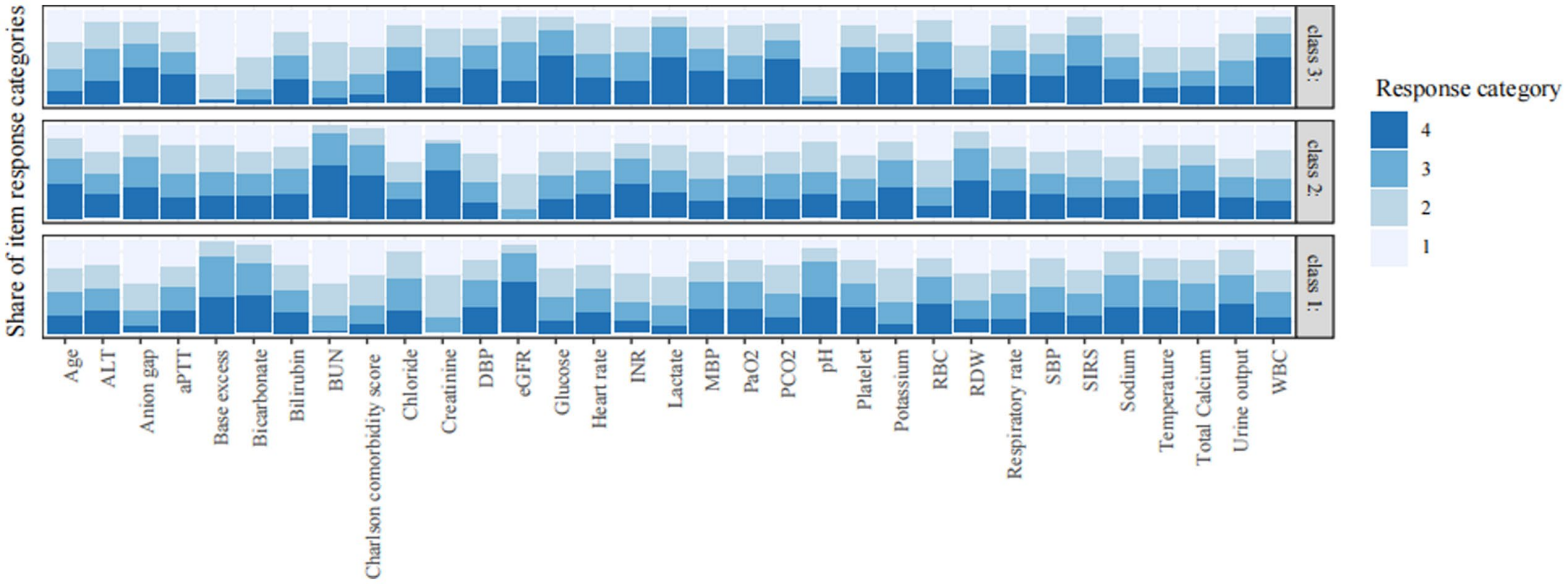
Characteristics of classes identified by latent class analysis. The response category of 1 to 4 is the quartile category by cutting continuous variables into four quartile categories. Category 1 refers to the lowest value and category 4 is the highest value. The vertical axis is the proportion of each response category. SBP, systolic blood pressure; DBP, diastolic blood pressure; MBP, mean blood pressure; WBC, white blood cell; RBC, red blood cell; RDW, red blood cell distribution width; BUN, blood urea nitrogen; eGFR, estimated glomerular filtration rate; PaO_2_, arterial oxygen partial pressure; PCO_2_, partial pressure of carbon dioxide; aPTT, activated partial thromboplastin time; INR, international normalized ratio; SIRS, systemic inflammatory response syndrome; ALT, alanine aminotransferase.

The underlying phenotypes were also verified by using LCA. As shown in Figure 3 and Supplementary Table S2, the best number of profiles was three as judged by entropy. Consistent with the result obtained by LPA, the LCA identified three profiles of CS: profile 1 was considered as the baseline group; profile 2 was characterized by advanced age, more comorbidities, and worse renal function; and profile 3 was characterized by SIRS-related indexes and acid–base balance disturbance.

### Vasopressor use and outcome

Patients in profile 3 showed a high incidence of the use of vasopressors (dopamine use: 36.5%; epinephrine use: 24.1%; norepinephrine use: 73.5%) were, as expected, more prevalent compared to the other two profiles (Table 2). In the whole cohort, patients receiving vasopressors were at higher risk of both ICU (OR: 3.36, 95% CI: 2.12–5.35, *p* < 0.001) and hospital mortality (OR: 1.99, 95% CI: 1.36–2.90, *p* < 0.001) (Table 3). Vasopressor agents were associated with improved risk of hospital death for profile 2 (OR: 2.03, 95% CI: 1.15–3.60, *p* = 0.015) and profile 3 (OR: 2.91, 95% CI: 1.02–8.32, *p* = 0.047).

## Discussion

### Major findings

CS is a clinical condition that results from ventricular failure due to acute coronary ischemia eventually leading to inadequate peripheral tissue perfusion, tissue and cellular ischemia, end-organ damage and multiorgan system failure (3). Using routinely collected clinical data in a large electronic database, this study identified 3 profiles of CS: profile 1 was the baseline group characterized by the lowest mortality rate; profile 2 was characterized by advanced age, comorbidities, worse renal function, and the second highest mortality; and profile 3 was characterized by SIRS-related indexes and acid–base balance disturbance, and the highest mortality rate. Vasopressor use was associated with an improved risk of in-hospital and ICU mortality for profile 2 and profile 3, respectively, after adjustment for multiple underlying confounders. The results of vasopressor use showed no significance for profile 1.

### Relation to other works and interpretation of the findings

Several prognostic classifications or risk stratifications of CS have been reported. For example, based on cardiac output (i.e., inadequate [cold] versus adequate [warm]) and volume status (i.e., overloaded [wet] versus euvolemic [dry]), CS patients are typically divided into four phenotypes (33, 34). Additionally, the IABP-SHOCK II score has three risk categories comprised of six variables and a maximum of nine points (17). Patients in the low, intermediate, and high-risk categories have an in-hospital mortality risk of 20–30%, 40–60%, and 70–90%, respectively. The Society of Cardiovascular Angiography and Interventions (SCAI) staging, which describes the stages of CS from A to E, offers the possibility of discriminating between morbidity and mortality (12). It can be used to track the severity of shock over the course of a hospital stay. This classification system can be used to monitor the severity of shock over the duration of a hospital stay. However, it was noted that some of these classification tools are based on expert consensus and theoretical considerations rather than on clinical evidence. To avoid complexity, some of these classifications contain only a few characteristics and depend on specific, although arbitrary, cutoff values that could result in bias and fail to capture the full variability of patient profiles. Additionally, some continuous variables in the classification were changed into a categorized variable, which might cause a loss of information on between-subject variability.

In the current study, three distinct profiles of critically ill patients with CS after AMI were determined by using 33 clinical variables obtained from electronic health record (EHR) and the LPA, a kind of unsupervised machine learning technique. The main difference between LPA and other clustering algorithms (such as latent variable mixture modeling, K-means clustering, and LCA) is that LPA uses a “model-based clustering” method to derive clusters from a probabilistic model of the data’s distribution (28, 35–37). Therefore, LPA fits a model that describes the distribution of the data, and you evaluate probabilities of particular patients being members of particular latent profiles based on this model rather than searching for clusters with some arbitrarily chosen distance measure. Furthermore, we had presumed that the structure of our data was the result of some processes or “latent structure.” Since LPA enabled us to model the latent structure underlying the data, it seemed like a good option.

Profile 3, characterized by SIRS-related indexes and acid–base balance disturbance, showed the highest all-cause in-hospital and ICU mortality rate. SIRS is frequently observed among patients with AMI or CS and is considered a major pathophysiologic mechanism contributing to worsening shock and organ injury leading to adverse outcomes (38). Jentzer et al. (38) found that one-third of cardiac ICU patients meet clinical criteria for SIRS at the time of admission, and these patients have higher illness severity and worse outcomes across the spectrum of SCAI shock stages. In conclusion, SIRS can increase the mortality in patients with CS by exacerbating oxidative stress, pro-inflammatory cytokines, coagulopathy, impaired tissue perfusion, and the risk of infections. Early recognition and prompt treatment of SIRS are crucial to prevent or mitigate its detrimental effects and patient outcomes. Electrolyte and acid–base balance disorders are also important factors affecting patient prognosis. Several studies have demonstrated that a composite laboratory assessment of acidosis (including lactate, pH, anion gap, and base excess) was highly associated with short-term mortality in patients with CS (17, 39, 40). Profile 2 was characterized by advanced age, comorbidities, worse renal function, and the second highest mortality. It is noteworthy that there is some overlap in clinical characteristics between profile 2 and profile 3 patients. For example, worse kidney function of profile 2 often leads to acid–base disorders and the associated disruption of electrolyte balance, which overlaps with the characteristics of profile 3. However, the impairment of renal function can also lead to an increase in fluid overload, which can further aggravate the patient’s hemodynamic instability (41). In addition, renal dysfunction itself can cause an accumulation of uremic toxins and inflammation, which can further exacerbate the CS patient’s condition (42).

Vasopressors are commonly used in the management of patients with CS. This study found that the use of vasopressors was associated with a higher risk of hospital mortality in the whole cohort, profile 2, and profile 3, respectively. However, it was noted that for the patients who did not use vasopressors, the physical signs and laboratory test data are better than those who did. Additionally, vasopressors could increase myocardial oxygen consumption, reduce blood flow to peripheral organs, which might impair microcirculation, and increase cardiac afterload and the risk of arrhythmias. It is important to note that the use of vasopressors can be life-saving in some cases of CS, and their benefits should be weighed against the potential risks on a case-by-case basis. Additionally, other factors such as the timing and dosing of vasopressors may also play a role in their association with increased mortality (43).

### Clinical implications

The goal of precision medicine is to personalize healthcare with medical decisions, procedures, treatments, or products that are tailored to each patient. A small step toward precision medicine may be the identification of the subphenotypes of ICU patients within a specific diagnostic group. The current research, to the best of our knowledge, is the first to investigate CS subphenotypes using LPA and clinical variables acquired from an electronic health record, which would make it easier to apply the findings to routine clinical practice, and be generalizable to other institutions. In addition, we strictly limited the etiology of CS to avoid the influence of different causes on the research results. Subphenotypes of a disease are usually explored by using genomic information or biomarkers that were not routinely obtained in clinical practice. The identified profiles of CS in the current study may be used and estimated by clinicians in the ICU to quickly assess patients with CS and enhance clinical decision-making, as the key features identified in this study are rapid, easy, and inexpensive laboratory tests. Instead of aiming for a one-size-fits-all solution, these clusters may help clinical trials by developing treatment strategies that are tailored to a CS phenotype. This could pave the way for more individualized health care. Summarily, CS patients exhibit significant heterogeneity, and identification of subphenotypes might help to stratify patients who are most likely to benefit from potential therapies in a clinical trial and facilitate the subsequent randomized controlled trial designs.

### Limitation

This study has a few limitations that need to be acknowledged. First, all of the patients who participated in this research came from the MIMIC-III database, so all the data collected in this single-centre retrospective study are not as precise as the dataset analysis collected in prospective cohort study. Second, although it is reasonable to limit the variables used in modeling to those found in clinical practice, this may make it harder to separate profiles. Utilizing genomics and biomarkers together would be preferable. However, the database did not contain any biomarkers that are not typically obtained. Third, this study only discussed three common vasoconstrictor drugs. Other types of vasopressors and their dosages were not discussed because there was limited relevant data in the database. Fourth, instead of providing a definitive profile membership, LPA assigns the profile with the highest posterior probability and provides posterior probabilities for each profile. As a result, the membership status of the profile is unsure. Fifth, our classification tool cannot dynamically evaluate the severity and progression of CS because it only enrolled the CS variable at an early stage. As a result, we will examine the relationship between phenotypes and endpoints within each SCAI stage to characterize the progression of disease severity throughout a hospital stay. Sixth, since the data come mainly from patients in the United States, more clinical analysis is needed to testify whether our results are applicable to the patients with CS in China. Lastly, only the initial test results were taken into account for subsequent analyses if patients had received multiple measurements of their vital signs or laboratory tests, which might not reflect the most serious condition of CS patients.

## Conclusion

There were three distinct CS phenotypes identified in this study, which showed different mortality outcomes and responses to vasopressor use.

## Data availability statement

The original contributions presented in the study are included in the article/Supplementary material, further inquiries can be directed to the corresponding authors.

## Ethics statement

The establishment of the MIMIC-IV database was approved by the Massachusetts Institute of Technology and the Institutional Review Board of Beth Israel Deaconess Medical Center (BIDMC) (24). Prior to study analyses, all data were made anonymous, so informed consent was not necessary.

## Author contributions

YY, JR, YZ, and ZW conceived the analysis and co-wrote the paper. YY, JX, YZ, and QX extracted all the data. ZW, YY, WX, and PW undertook and refined the inclusion process. YY, JR, JX, and QX undertook the statistical analyses. YY, YZ, and ZW were consulted for clinical issues. All authors contributed to and revised the final manuscript.

## Funding

This work was supported by the National Nature Science Foundation of China (No. 81770244), Medical Science and Technology Youth Cultivation Plan (Nos. 17QNP013 and 20QNPY038), Shanghai Municipal Commission of Science and Technology (No. 17ZR1439100), Shanghai Shenkang Medicine Developing Project (No. SHDC12014107), and Shanghai Science and Technology Committee Medicine Leading Project (No. 15411960100).

## Conflict of interest

The authors declare that the research was conducted in the absence of any commercial or financial relationships that could be construed as a potential conflict of interest.

## Publisher’s note

All claims expressed in this article are solely those of the authors and do not necessarily represent those of their affiliated organizations, or those of the publisher, the editors and the reviewers. Any product that may be evaluated in this article, or claim that may be made by its manufacturer, is not guaranteed or endorsed by the publisher.

## Abbreviations

CS: cardiogenic shock
AMI: acute myocardial ischemia
LPA: latent profile analysis
MIMIC: Medical Information Mart for Intensive Care
ICU: intensive care unit
BIDMC: Institutional Review Board of Beth Israel Deaconess Medical Center
ICD: International Classification of Diseases
SBP: systolic blood pressure
DBP: diastolic blood pressure
MBP: mean blood pressure
WBC: white blood cell
RBC: red blood cell
RDW: red blood cell distribution width
BUN: blood urea nitrogen
eGFR: estimated glomerular filtration rate
PaO_2_: arterial oxygen partial pressure
PCO_2_: partial pressure of carbon dioxide
aPTT: activated partial thromboplastin time
INR: international normalized ratio
SIRS: systemic inflammatory response syndrome
IABP: intra-aortic balloon pump
RRT: renal replacement treatment
LOS: length of stay
BIC: Bayesian information criteria
BLRT: bootstrap likelihood ratio test
LCA: latent class analysis
SD: standard deviation
IQR: interquartile range
LR: logistic regression
OR: odds ratio
CI: confidence interval
SCAI: Society of Cardiovascular Angiography and Interventions
EHR: Electronic health record
