# Supplementary material for: Phenotyping cardiogenic shock that showed different clinical outcomes and responses to vasopressor use: a latent profile analysis from MIMIC-IV database
Source: Front Med (Lausanne). 2023 Jun 22;10:1186119. doi: 10.3389/fmed.2023.1186119 (PMC10325854; doi:10.3389/fmed.2023.1186119)
Supplement: Supplementary file 1 [file Table_1.docx]

**Table S1. Latent profile analysis for choosing the best number of profiles**

| **Number of classes** | **AIC** | **CAIC** | **BIC** | **SABIC** | **Entropy** | **Number of patients in each latent profile** | | | | | | | | ***P*** |
| --- | --- | --- | --- | --- | --- | --- | --- | --- | --- | --- | --- | --- | --- | --- |
|  |  |  |  |  |  | **1** | **2** | **3** | **4** | **5** | **6** | **7** | **8** |  |
| **2** | 67,445.73 | 68,032.62 | 67,926.62 | 67,590.06 | 0.81 | 365 | 325 |  |  |  |  |  |  | 0.009 |
| **3** | 66,652.36 | 67,438.57 | 67,296.57 | 66,845.70 | 0.84 | 259 | 261 | 170 |  |  |  |  |  | 0.009 |
| **4** | 66,652.31 | 67,637.84 | 67,459.84 | 66,894.67 | 0.88 | 261 | 169 | 259 | 1 |  |  |  |  | 0.009 |
| **5** | 66,643.81 | 67,828.66 | 67,614.66 | 66,935.17 | 0.89 | 261 | 169 | 258 | 1 | 1 |  |  |  | 0.009 |
| **6** | 66,664.05 | 68,048.22 | 67,798.22 | 67,004.43 | 0.90 | 260 | 170 | 257 | 1 | 1 | 1 |  |  | 0.059 |
| **7** | 66,680.15 | 68,263.64 | 67,977.64 | 67,069.55 | 0.91 | 260 | 169 | 257 | 1 | 1 | 1 | 1 |  | 0.019 |
| **8** | 66,693.49 | 68,476.30 | 68,154.30 | 67,131.90 | 0.92 | 258 | 170 | 257 | 1 | 1 | 1 | 1 | 1 | 0.029 |

**Abbreviations:** AIC Akaike information criterion, BIC Bayesian information criteria, CAIC consistent Akaike information criterion, SABIC sample size adjusted Bayesian information criteria.

**Table S2. choosing the best number of classes by using latent class analysis**

|  | **log_likelihood** | **df** | **BIC** | **aBIC** | **cAIC** | **likelihood_ratio** | | **Entropy** | **N of classes** |
| --- | --- | --- | --- | --- | --- | --- | --- | --- | --- |
| **1** | -32713.194 | 479 | 66805.630 | 66135.671 | 67016.63 | | 56405.754 | 0.887 | 2 |
| **2** | -32233.737 | 373 | 66539.605 | 65533.079 | 66856.605 | | 55446.839 | 0.909 | 3 |
| **3** | -31984.36 | 267 | 66733.741 | 65390.648 | 67156.741 | | 54948.086 | 0.909 | 4 |
| **4** | -31749.722 | 161 | 66957.354 | 65277.695 | 67486.354 | | 54478.81 | 0.912 | 5 |
| **5** | -31584.195 | 55 | 67319.189 | 65302.963 | 67954.189 | | 54147.756 | 0.904 | 6 |
| **6** | -31424.259 | -51 | 67692.206 | 65339.413 | 68433.206 | | 53827.883 | 0.948 | 7 |
| **7** | -31282.802 | -157 | 68102.183 | 65412.823 | 68949.183 | | 53544.971 | 0.908 | 8 |

**Abbreviations:** AIC Akaike information criterion, BIC Bayesian information criteria, CAIC consistent Akaike information criterion, SABIC sample size adjusted Bayesian information criteria.
